# Potent neutralization and therapeutic efficacy of bovine rotavirus-specific VHH antibodies in infected calves

**DOI:** 10.1186/s13567-026-01765-3

**Published:** 2026-05-21

**Authors:** Qihuan Zhao, Min Gao, Puchen Li, Bo Wang, Baohui Li, Gege Rile, Chengquan Du, Jingjing Wang, Hui Wang, Jingsi Mei, Shujun Zhang, Fuxiang Bao

**Affiliations:** 1 College of Veterinary Medicine, Inner Mongolia Agricultural University, No. 306, Zhaowuda Road, Saihan District, Huhhot, 010018 China; 2 Fisheries Technology Division, Inner Mongolia Autonomous Region Agricultural and Animal Husbandry Technology Promotion Center, Huhhot, 010010 China; 3 Wuyuan County Animal Disease Prevention and Control Center, Bayannur, 015100 China; 4 Key Laboratory of Clinical Diagnosis and Treatment Techniques for Animal Disease, Ministry of Agriculture and Rural Affairs, Huhhot, 010010 China

**Keywords:** Bovine rotavirus, single-domain antibodies, treatment

## Abstract

Bovine rotavirus is one of the main pathogens causing acute diarrhea in calves under 1 month of age, posing a serious threat to intensive cattle farming, and there are no effective therapeutic agents owing to the mixed infection of bovine rotavirus (BRV) with other pathogens. In this study, we successfully constructed a phage display antibody library by immunizing Bactrian camels, with a capacity of 6.5 × 10^8^ and a recombinant positive rate of 81.25%. Three high-activity variable heavy-chain domains (VHH) were obtained by using BRV as the antigen, and the gene fragments were ligated with the prokaryotic expression vector pET–22b(+). The recombinant VHH was successfully expressed under induction by isopropyl β-d-1-thiogalactopyranoside (IPTG). In addition, the recombinant VHH could specifically bind to BRV in an enzyme-linked immunosorbent assay (ELISA) and cellular immunofluorescence assays. The neutralizing activity of VHH was further confirmed by a virus neutralization test. Furthermore, our data showed that recombinant VHH could alleviate symptoms such as diarrhea and high fever, and reduce the amount of virus release in a BRV-challenged calf model. These results suggest that the single-domain antibodies against BRV screened in this study offer an effective treatment in BRV infection, facilitating the development of novel therapeutics for BRV.

**Supplementary Information:**

The online version contains supplementary material available at 10.1186/s13567-026-01765-3.

## Introduction

Diarrhea in neonatal calves represents a major challenge to the cattle industry, with a complex and often multifactorial etiology [1]. Group A bovine rotavirus (BRV) is the primary pathogen responsible for rotavirus infection in domestic livestock. Bovine rotavirus is a primary causative agent of diarrhea in newborn calves and frequently co-infects with other enteric pathogens, thereby exacerbating disease severity and significantly increasing mortality risk [2]. Among calves younger than 6 weeks of age—particularly those within the first 2 weeks of life, which represent the most susceptible population—the mortality rate associated with rotavirus infection typically ranges from 5% to 20%. However, in uncontrolled outbreak situations, mortality in neonatal calves may reach as high as 80%, posing a serious threat to intensive cattle production systems [3].

BRV is a non-enveloped double-stranded RNA virus with an icosahedral symmetry [4]. In accordance with the recently revised nomenclature system of the International Committee on Taxonomy of Viruses (ICTV), its taxonomic position has been reclassified from the former family Reoviridae to the family Sedoreoviridae [6]. The virion has a diameter of approximately 70 nm and consists of a triple-layered protein capsid. Its structural components include the core proteins VP1, VP2, and VP3, an inner capsid layer composed of VP6, and an outer capsid layer formed by VP4 and VP7 [6]. According to the nucleotide sequences of the outer capsid proteins VP7 (G genotype) and VP4 (P genotype), BRV is classified into different genotypes, and these two proteins are the principal determinants of viral antigenicity and neutralization specificity [7]. To date, 12 G genotypes and 11 P genotypes have been identified worldwide. Among them, G6, G8, and G10 are the most prevalent G genotypes, whereas P [1], P [5], and P [11] are the predominant P genotypes. The most common genotype combinations include G6P [1], G6P [5], G6P [11], G8P [1], and G10P [11] [8]. In China, the distribution of BRV genotypes shows clear regional variation. G6P [1] predominates in northwestern China, whereas G6P [5] is the dominant genotype in northeastern China. In eastern China (e.g., Shandong Province), G6P [1] and G6P [11] are more frequently detected, while in southwestern China (e.g., Sichuan Province), co-circulation of G6P [1] and G8P [1] has been reported [9]. Overall, G6-associated genotypes are widely distributed across multiple regions in China.

Cross-protection among different BRV genotypes mainly depends on immune responses directed against VP7 and VP4. VP7 acts as the major neutralizing antigen and induces type-specific antibodies, whereas VP4 is cleaved into VP5 and VP8 during viral entry and mediates host cell attachment and penetration, thereby also inducing neutralizing antibodies. Consequently, VP7- and VP4-specific neutralizing antibodies constitute the fundamental basis of protective humoral immunity [7]. However, substantial antigenic variation exists among different genotypes, which limits antibody cross-neutralization. Immunity induced by a single genotype often fails to fully prevent infection with heterologous strains; therefore, its clinical significance is mainly reflected in reducing disease severity, such as decreasing viral shedding. The virus infects and damages the epithelial cells of the intestinal villi, with diarrhea occurring most frequently during the first 2 weeks after birth. Clinical manifestations range from mild diarrhea to severe dehydration, and severe cases—most commonly observed during this high-risk age period—may result in pronounced weakness and even death [10].

The control of BRV remains challenging owing to several significant limitations. To date, no specific antiviral therapy is available for BRV infection, and clinical management primarily relies on supportive treatment. In addition, highly efficacious and BRV-specific vaccines are not yet widely available in China. Commercially available vaccines are predominantly multivalent formulations, including either inactivated or live-attenuated preparations, which commonly incorporate antigens from other enteric pathogens such as bovine coronavirus and *Escherichia coli* (*E. coli*) [11]. These vaccines are mainly administered to pregnant cows to enhance colostral antibody levels, thereby providing passive immunity to neonatal calves. However, this strategy is inherently dependent on adequate maternal antibody transfer, and the resulting protection is transient, largely influenced by colostrum quality and intake, and incapable of inducing active and durable immune memory in calves [11]. Furthermore, multivalent formulations may give rise to antigenic interference, potentially compromising the magnitude of BRV-specific immune responses. In addition, both inactivated and live-attenuated vaccines often exhibit limited durability of immunity, frequently requiring booster immunizations to maintain adequate protection. Collectively, these factors restrict the long-term effectiveness of current BRV vaccination strategies in intensive cattle production systems.

Given these constraints, passive immunization has emerged as an important strategy for the prevention and control of BRV infection. Owing to their high affinity and antigen specificity, antibodies have become essential tools in the diagnosis and treatment of a wide range of diseases. Nevertheless, there is still no single, highly effective therapeutic antibody specifically targeting BRV [12].

In addition to conventional antibodies, camelids and cartilaginous fish possess a distinctive class of antibodies composed solely of heavy chains, lacking light chains. The antigen-binding fragment of these heavy-chain-only antibodies consists of a single variable domain, known as single-domain antibodies (sdAbs) [13–15]. These sdAbs, also referred to as nanobodies or variable heavy-chain domains (VHH), have a molecular weight of approximately 12–15 kilodalton (kDa), making them substantially smaller than conventional immunoglobulins. Owing to their unique structural characteristics, sdAbs exhibit high antigen-binding affinity and specificity, as well as enhanced solubility, hydrophilicity, and physicochemical stability. These advantageous properties have facilitated their widespread application in diagnostics, therapeutics, and biotechnology [16, 17].

In previous studies, we constructed a phage display antibody library through calf immunization and identified ultralong CDR H3 antibodies that showed both prophylactic and therapeutic potential against BRV infection in cattle [18]. Other sdAbs with distinct specificities have also been developed, which can be engineered as bispecific nanobodies for targeting tumor cells or employed as virus-specific binding reagents in diagnostic and purification applications [19]. Building on the inherent advantages of nanobodies, and the growing limitations on antibiotic use in livestock production, we sought to develop a nanobody capable of precise viral neutralization, thereby providing a new therapeutic alternative against BRV.

Therefore, the present study aimed to construct a BRV-specific sdAbs phage display antibody library and screen for sdAbs exhibiting high affinity and neutralizing activity. Bactrian camel was immunized with BRV antigens to generate the library, from which BRV-specific sdAbs were isolated and characterized. Their binding and neutralization capacities were systematically evaluated to determine their potential as an effective antibody-based therapeutic. This work seeks to establish a promising treatment strategy against BRV infection and to support the future development of passive immunotherapeutics.

## Materials and methods

### Serum titer determination and neutralization assay

A healthy 1-year-old female Bactrian camel was selected for immunization. Bovine rotavirus (rotavirus A isolate NM06, GenBank: PV996889.1–PV996900.1) used for this study was kindly provided by Professor Weiguang Zhou of the College of Veterinary Medicine, Inner Mongolia Agricultural University. After centrifugation of the BRV culture supernatant to remove cell debris, Bactrian camels were immunized via multipoint subcutaneous injection. For the primary immunization, 2 mL of BRV virus suspension (50% tissue culture infectious dose [TCID_50_]: 10^−7.75^/100 µL of the BRV strain NM06) was thoroughly emulsified with an equal volume of sterilized Freund’s Complete Adjuvant (Solarbio Life Sciences, Beijing, China) and inoculated. Booster immunizations were subsequently administered at 2-week intervals, using Freund’s Incomplete Adjuvant (Solarbio Life Sciences, Beijing, China) instead to mix with BRV as antigen. A total of four immunizations were administered, and 1 week after each immunization, blood was collected for serum separation to monitor antibody titers, and the serum obtained after the final immunization was used for the virus neutralization test. Prior to immunization, jugular vein blood was collected to obtain pre-immune serum for subsequent use. BRV with a TCID_50_ of approximately 10^−7.75^/100 µL was diluted 100-fold and used to coat 96-well enzyme-linked immunosorbent assay (ELISA) plates, which were incubated overnight at 4 °C. On the following day, the plates were blocked with 3% bovine serum albumin (BSA) for 2 h at room temperature and then washed thoroughly. Serum collected before the primary immunization served as the negative control, while phosphate-buffered saline (PBS) was used as the blank control. The antiserum obtained after immunization was subjected to twofold serial dilutions in PBS, from 1:1000 to 1:4 096 000, with two replicate wells for each dilution, and 100 μL of the diluted antiserum was added to each well, followed by incubation at 37 °C for 1 h. Subsequently, horseradish peroxidase (HRP)-conjugated goat anti-alpaca immunoglobulin G (IgG; 1:10 000) (NBbiolab, Chengdu, China) was added to the wells. After incubation for 1 h, TMB substrate (Solarbio Life Sciences, Beijing, China) was added, followed by the stop solution. The absorbance at 450 nm was measured to evaluate the antibody titers. Using the optical density at 450 nm (OD_450_) value of pre-immune serum (negative control) as the baseline, samples with an OD value ≥ 2.1 times the baseline were considered positive.The antibody titer was defined as the highest serum dilution above the cutoff [20]. For the serum antibody neutralization test, peripheral blood was collected from camels after the final immunization. Serum samples were separated and heat-inactivated at 56 °C for 30 min prior to testing. Twofold serial dilutions of the serum samples were prepared in 96-well plates (50 μL per well). Each dilution was mixed with an equal volume (50 μL) of BRV suspension containing 100 TCID_50_ of virus. Virus control groups containing 0.1, 1, 10, and 100 TCID_50_ were included to confirm assay validity. The virus–serum mixtures were incubated at 37 °C for 1 h to allow neutralization. Subsequently, 100 μL of the mixture was added to confluent MA104 cell monolayers in 96-well plates. The cells were cultured in an incubator at 37 °C with 5% CO_2_ for 3–5 d. Each dilution was tested in triplicate wells, and the experiment was independently repeated twice. Cytopathic effects (CPE) were observed daily under an inverted microscope. Wells showing ≥ 50% cell rounding, detachment, or lysis compared with cell control wells were considered CPE-positive. The neutralization titer was defined as the reciprocal of the highest serum dilution that inhibited 50% of virus-induced CPE. The 50% endpoint was calculated using the Reed–Muench method [21]. After the final immunization, peripheral blood mononuclear cells (PBMCs) were also isolated for library construction. The camel was housed at the Laboratory Animal Center of the College of Veterinary Medicine, Inner Mongolia Agricultural University with ad libitum access to food and water. All experimental procedures were approved by the Animal Care and Use Committee of Inner Mongolia Agricultural University and conducted in compliance with institutional and national guidelines.

### Construction and screening of a BRV-specific phage display library

PBMCs were isolated from whole blood using a Bovine Peripheral Blood Lymphocyte Isolation Kit (Tianjin Haoyang Biological Products Technology Co., Ltd., Tianjin, China). Subsequent experiments were performed on the basis of previous experience in our laboratory and methods described in the literature [22]. Total RNA was extracted from PBMCs using TRIzol reagent (Ambion, Austin, TX, USA) according to the manufacturer’s instructions. The extracted RNA was directly subjected to one-step reverse-transcription polymerase chain reaction (RT-PCR) amplification using the PrimeScript™ One Step RT-PCR Kit (TaKaRa, Shiga, Japan), which allows reverse transcription and PCR amplification to be conducted in a single reaction. The primer sequences used in this study are listed in Table 1. Primers were designed on the basis of our laboratory’s previous work, and amplification was performed according to a method described in the literature [23]. In the first round of PCR, primers P1 and P2 were used to amplify camel IgG heavy-chain antibody-related fragments, generating two products of approximately 900 base pairs (bp) (VH–CH1–CH2) and 600 bp (VHH–CH2). The PCR products were separated by agarose gel electrophoresis. The 600-bp VHH–CH2 fragment was excised and purified using a Gel Extraction Kit (Tiangen, Beijing, China), and used as the template for the second round of PCR. This fragment was then amplified with primers VHH–IF and VHH–IR to obtain the final VHH gene product. Subsequently, a phage antibody library was constructed following a method described in the literature [19]. The purified VHH PCR product and the pMECS plasmid vector (kindly provided by Professor Serge Muyldermans, Vrije Universiteit Brussel, Belgium) were digested with the restriction endonucleases *Nco* I and *Not* I. The digested fragments were ligated using T4 DNA ligase (Takara Bio, Shiga, Japan) to construct the recombinant pMECS–VHH plasmid. The resulting plasmid was then introduced into electrocompetent *E. coli* TG1 cells (GE Healthcare, Chicago, IL, USA) by electrotransformation to establish the primary antibody library. The library capacity was determined, and the insertion rate of the VHH gene in the recombinant plasmids was verified by PCR using the sequencing primers MP57 and GIII. Colony PCR verification: Colony PCR was performed using primers MP57 and GIII in a 25-μL reaction system. The thermal cycling conditions were as follows: 95 °C for 5 min; 30 cycles of 95 °C for 30 s, 55 °C for 30 s, and 72 °C for 1 min; and a final extension at 72 °C for 10 min.

**Table 1:** **PCR primers**

Primer	Sequence
P1	5′-GGACTCGGCCACMTAYTACTG-3′
P2	5′-GCTCGAGACGGTGAYCAG-3′
VHH–IR	5′-AGTTGTTCCTTCTATGCGGCCCAGCCGGCCATGGCTGAKGTBCAGCTGGTGGAGTCTGG-3′
VHH–IF	5′-ATTGCGTCAGCTATTAGTGCGGCCGCTGAGGAGACRGTGACCWGGGTCC-3′
T7	5′-TAATACGACTCACTATAGGG-3′
T7t	5′-TGCTAGTTATTGCTCAGCGG-3′
MP57	5′-TTATGCTTCCGGCTCGTATG-3′
GIII	5′-CCACAGACAGCCCTCATAG-3′

A 1-mL aliquot of the phage antibody library was inoculated into 100 mL of 2× YT medium and cultured at 37 °C with shaking at 250 rpm for 2.5 h. M13K07 helper phage (NBbiolab, Chengdu, China) was added to the culture, followed by incubation at 37 °C with shaking at 250 rpm for 1 h. The mixture was then centrifuged at 2000 *g* and 4 °C for 10 min. The pellet was resuspended in 40 mL of 2× YT–AK medium (containing ampicillin and kanamycin) and incubated overnight at 37 °C with shaking at 250 rpm. The following day, the culture was centrifuged at 7197 *g* and 4 °C for 25 min. The supernatant was collected and mixed with an equal volume of 3% bovine serum albumin (BSA), followed by incubation at 4 °C for 10 min. Then, 8 mL of 20% PEG–NaCl was added, and the mixture was incubated at 4 °C for 2 h. After centrifugation at 7197 *g* and 4 °C for 30 min, the precipitated phage particles were resuspended in 1 mL of PBS to obtain the amplified recombinant phage library.

For biopanning, the phage library was introduced into an immunotube coated with 5 mL of BRV (TCID_50_: 10^−7.75^/100 µL) and incubated in a 37 °C water bath for 1 h. The tube was washed to remove unbound phages, and the specifically bound recombinant phages were eluted and used to infect 3 mL of log-phase *E. coli* TG1 cells (OD_600_ ≈ 0.6) in a water bath for 30 min. Subsequently, a 200-μL aliquot of the infected culture was spread onto 2× YT–AG solid medium (supplemented with 20% glucose) and incubated overnight at 37 °C. After three rounds of such screening, 92 single colonies were randomly selected from the third-round output and inoculated into 2× YT–AG liquid medium for overnight culture at 37 °C with shaking at 250 rpm. A 50-μL aliquot of each culture was then transferred to a fresh tube containing 400 µL of 2× YT–AG medium and M13K07 helper phage, followed by incubation at 37 °C with shaking at 250 rpm for 2 h. The cells were collected by centrifugation at 12 000 *g* for 5 min, resuspended in 400 µL of 2× YT–AK liquid medium, and cultured overnight under the same conditions. The next day, 400 µL of the culture supernatant was mixed with an equal volume of 20% PEG–NaCl and incubated at 4 °C for 30 min to precipitate the phage particles.

The recombinant phages were incubated in an ELISA plate precoated with BRV (TCID_50_: 10^−7.75^/100 µL) for 2 h at room temperature. The M13K07 helper phage and PBS buffer were used as the negative control and blank control, respectively. After washing, 100 µL of horseradish peroxidase (HRP)-conjugated anti-M13 monoclonal antibody (AlpSdAbs VHH, Chengdu, China), diluted at 1:5000, was added to each well and incubated for 1 h at room temperature. Subsequently, 100 µL of TMB substrate solution (Solarbio Life Sciences, Beijing, China) was added and allowed to develop for 10 min. The reaction was stopped, and the absorbance was measured at 405 nm using a microplate reader. A positive binding signal was defined as a sample-to-negative (P/N) absorbance ratio of ≥ 2.1.

### Cloning, expression, and purification of BRV-specific sdAbs

The pMECS–VHH recombinant plasmid and the pET–22b(+) vector carrying a His tag (maintained in the Public Health Laboratory of the College of Veterinary Medicine, Inner Mongolia Agricultural University) were subjected to double digestion using *Nco* I and *Not* I restriction enzymes. The resulting VHH gene fragment was purified and ligated into the linearized pET–22b(+) plasmid using T4 DNA ligase. The ligation product was then transformed into *E. coli* BL21 (DE3) competent cells (Sangon Biotech, Shanghai, China). Positive transformants were cultured and induced with 1 mM isopropyl β-d-1-thiogalactopyranoside (IPTG) at 18 °C with shaking at 130 rpm for 12 h. After induction, the bacterial cells were harvested and lysed by ultrasonication. The lysate was separated by centrifugation into soluble (supernatant) and insoluble (pellet) fractions, which were subsequently analyzed by sodium dodecyl sulfate–polyacrylamide gel electrophoresis (SDS–PAGE) to evaluate the expression and solubility of the recombinant VHH antibody. The recombinant protein was purified using Ni–NTA Sefinose resin (Sangon Biotech, Shanghai, China) under native conditions. The eluted protein was again analyzed by SDS–PAGE to confirm purity and molecular weight. The protein expression and purification experiments were independently repeated three times.

### In vitro characterization of BRV-specific sdAbs

#### ELISA assay

The purified VHH antibody was subjected to a twofold serial dilution starting from 10 μg/mL. Each dilution was added to an ELISA plate precoated with BRV (TCID_50_: 10^−7.75^/100 µL) and incubated at 37 °C for 1 h. The supernatant from induced *E. coli* carrying the empty pET–22b(+) plasmid was used as the negative control, while PBS served as the blank control. After incubation and washing, an HRP-conjugated anti-6× His monoclonal antibody (Proteintech, Wuhan, China) diluted at 1:10 000 was added to each well, followed by incubation at 37 °C for 1 h. The plate was then developed with 100 μL of TMB substrate (Solarbio Life Sciences) for 15 min, and the absorbance was measured at 450 nm using a microplate reader to determine the binding activity of the VHH antibody. Each dilution was set up in duplicate wells, and the experiment was independently repeated three times.

#### Immunofluorescence assay

MA-104 cells were infected with BRV (TCID_50_: 10^−7.75^/100 µL) for 12 h, then fixed with 4% paraformaldehyde (Biosharp) for 20 min at room temperature. After fixation, the cells were blocked with 5% BSA for 1 h to minimize nonspecific binding. Subsequently, the cells were incubated with 2 mL of the purified VHH antibody (50 µg/mL) overnight at 4 °C. Following primary antibody incubation, the cells were stained with CoraLite^®^ Plus 488-conjugated anti-6× His tag antibody (Proteintech, Wuhan, China) at a 1:500 dilution for 1 h in the dark. Nuclei were counterstained with 4′,6-diamidino-2-phenylindole (DAPI) for 10 min. Fluorescence images were captured using a ZEISS LSM-800 confocal microscope (Oberkochen, Germany).

### Detection of neutralizing activity of BRV specific sdAbs

BRV (TCID_50_: 10^−7.75^/100 µL) was activated by treatment with trypsin at a final concentration of 0.25 µg/mL and incubation at 37 °C for 30 min. Subsequently, the activated virus was mixed with an equal volume of each test antibody diluted to 40, 20, 10, and 5 µg/mL respectively, followed by incubation at 37 °C for 1 h. The virus–antibody mixtures were then added to monolayers of MA-104 cells and cultured in a 37 °C, 5% CO_2_ incubator for 3–5 days. CPE were observed daily to evaluate the neutralizing activity of the antibodies.

### In vivo evaluation in a calf challenge model

#### Experimental design and clinical scoring

A group of 12 5-day-old neonatal male Holstein calves were obtained from a dairy farm in Hohhot, Inner Mongolia Autonomuos Region, China, and housed at the Laboratory Animal Center of the College of Veterinary Medicine, Inner Mongolia Agricultural University. These calves were randomly assigned to three groups (four calves per group): the BRV challenge group (calves 1–4, infected with the BRV NM06 strain), the antibody treatment group (calves 5–8), and the PBS control group (calves 9–12). All animals were fed with 2 L of milk replacer (Inner Mongolia Knight Dairy Group Co., Ltd., Baotou, China) twice daily. Calves in the BRV group and the antibody group were orally administered 10 mL of BRV (TCID_50_: 10^−7.75^/100 µL), whereas those in the PBS group received 10 mL of PBS. During the intervention phase, purified VHH clone 73 was dissolved in 500 mL of physiological saline and administered intravenously to calves in the antibody group at a dose of 1 mg/kg, twice daily for six consecutive days. Clinical observations were performed daily at fixed time points. Recorded parameters included rectal temperature, mental status, feeding behavior, and occurrence of diarrhea. Fecal samples were collected regularly and scored according to a standardized system (Additional file 1). At the end of the experiment, the calves were humanely euthanized by injection of saturated potassium chloride under deep anesthesia. All experimental procedures were carried out in accordance with the national and institutional guidelines for animal care and use, and were approved by the Animal Care and Use Committee of Inner Mongolia Agricultural University (approval no: NND2022023; approval date: 7 March 2022).

#### Viral load quantification by RT-qPCR

Viral RNA extraction and RT-qPCR were performed as previously described by Sun et al., [24]. Briefly, 0.2 g of each fecal sample was homogenized in 1 mL of PBS, subjected to three freeze–thaw cycles at −80 °C, and then filtered through a 0.22 μm membrane (Merck KGaA, Darmstadt, Germany). The filtrate was centrifuged at 12 000 *g* for 1 min, and the supernatant was used for RNA extraction with a Viral Genome DNA/RNA Extraction Kit (TIANGEN, Beijing, China). RT-qPCR was carried out using the following 25 μL reaction system: 12.5 μL of 2× One Step RT-PCR Buffer III, 0.5 μL of Ex-Taq HS, 0.5 μL of PrimeScript RT Enzyme Mix II, 1 μL of BRV-specific probe (10 μM), 1 μL each of forward and reverse primers BRV-F and BRV-R (10 μM) (Table 2), and 2 μL of template RNA. Nuclease-free water was added to adjust the final volume. The thermal cycling conditions consisted of reverse transcription at 42 °C for 5 min, initial denaturation at 95 °C for 10 s, followed by 45 cycles of denaturation at 95 °C for 5 s, and annealing/extension at 60 °C for 30 s. The quantification cycle (Cq) values were recorded, and absolute quantification was performed on the basis of a standard curve. Each sample was performed in duplicate for qPCR detection.

**Table 2:** **qPCR primers** [24]

Target gene	Primer/probe	Sequence (5′ → 3′)	Amplicon size (bp)	Tm value (°C)	Thermal cycling conditions
BRV *NSP5*	BRV-F	CATGTTGTCAAAGTCTCCAGA	127	53.3	Reverse Transcription: 42 °C for 5 min (1 cycle); initial denaturation: 95 °C for 10 s (1 cycle); amplification (45 cycles): 95 °C for 5 s, 60 °C for 30 s
	BRV-R	TGAATCCATAGACACGCCAGC		57.5	
	BRV-probe	ROX-CTGATTCTGCTTCAAACGATCCACTCACCAGC-BHQ2		64.4	

### Tissue sections

Small intestinal tissues were collected from calves in each experimental group and fixed in 4% paraformaldehyde (PFA) overnight at room temperature. The following day, fixed tissues were rinsed, dehydrated through a graded ethanol series, cleared in xylene, and embedded in paraffin according to standard histological procedures. Sections of 5 μm thickness were prepared using a rotary microtome (KD-202A, Hangzhou Kedi, China) and stained with hematoxylin and eosin (H&E; Solarbio, Beijing, China). Morphological examination was performed under a light microscope (NIKON ECLIPSE Ts2, Tokyo, Japan) to evaluate pathological changes, including intestinal epithelial cell damage, inflammatory infiltration, and vacuolation of villous structures.

### Statistical analysis

All statistical analyses were performed using GraphPad Prism 8.0 (GraphPad Software, Inc., La Jolla, CA, USA). Data are presented as the mean ± standard deviation from three independent replicates. Differences among multiple groups were assessed by one-way analysis of variance (ANOVA). A *p*-value of less than 0.05 was considered statistically significant (*p < 0.05), and a *p*-value of less than 0.01 was considered highly significant (**p < 0.01).

## Results

### Immune serum titer and neutralizing activity

**Figure 1:**
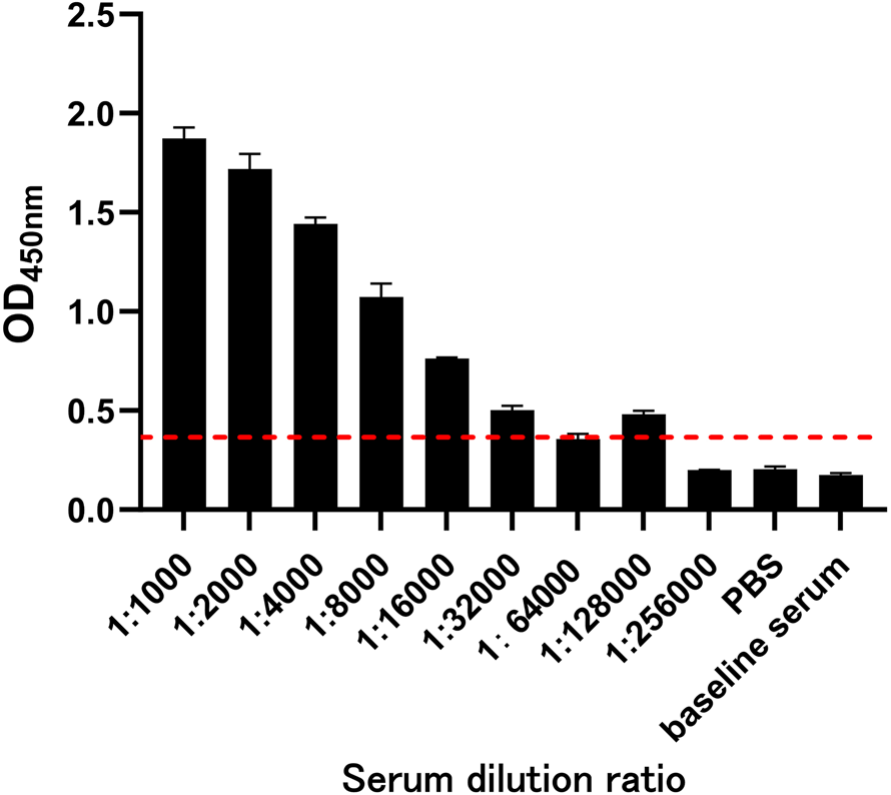
**Immune serum titers**. **A** Indirect ELISA was used to determine the antibody titers in camel sera after the fourth immunization. Goat anti-alpaca IgG (HRP) was used as the secondary antibody to detect the immune response. PBS served as the blank control, and pre-immune camel serum as the negative control. A reference line was set in the figure for result determination. Antibody titers were defined as the highest serum dilution that exceeded the reference line.

To evaluate the humoral immune response against BRV, serum samples were isolated from whole blood of the immunized Bactrian camel for the assessment of specific antibody production and virus-neutralizing activity. The baseline serum exhibited an OD_450_ nm value of 0.175, while the PBS control showed a value of 0.214. The antibody titer in the post-immunization serum reached 1:32 000, with an OD_450_ nm value 2.1-fold higher than that of the baseline serum, indicating a strong humoral immune response as shown in Figure 1. Using the Reed–Muench method, it was determined that a 1:21 dilution of the immune serum provided 50% protection against BRV-induced CPE in MA104 cells (Additional file 2).

### Construction and enrichment of the BRV-specific phage display library

**Figure 2:**
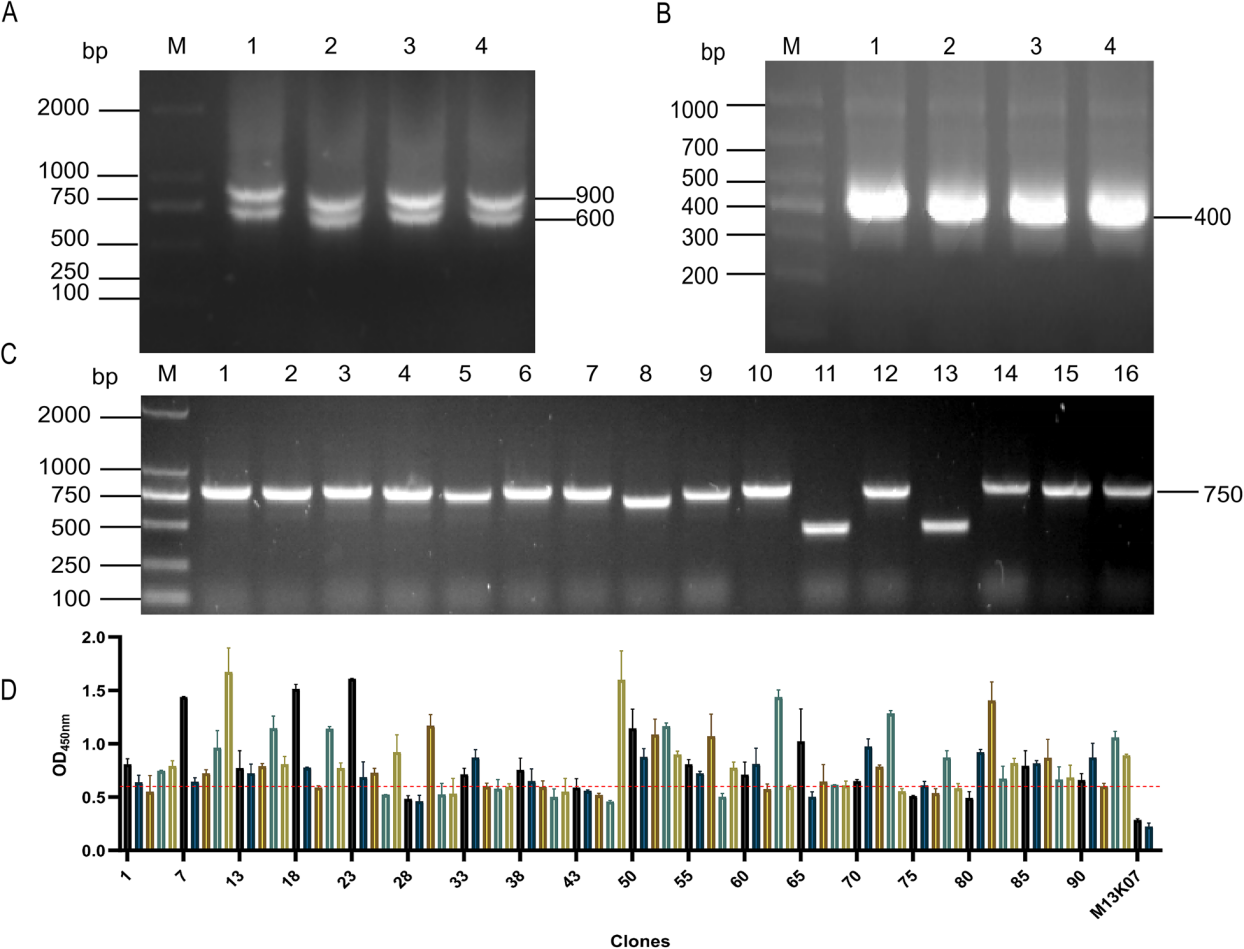
**Construction and screening of phage display library**. **A** PBMCs were isolated from camels, and RNA was extracted as a template for RT-PCR to amplify the gene sequence containing the leader signal sequence before the CH2 region. The amplification yielded two fragments: a 900-bp fragment (VH–CH1–CH2) and a 600-bp fragment (VHH–CH2). Lane M represents the DNA marker (2000–100 bp), and lanes 1–4 represent the amplified fragments. **B** The 600-bp fragment obtained from the first round of PCR was used as a template for the second round of PCR, which successfully generated the full-length VHH gene (spanning FR1 to FR4) with an approximate size of 400 bp. Lane M represents the DNA marker (1000–200 bp), and lanes 1–4 represent the amplified fragments. **C** A total of 16 clones were randomly selected from the constructed phage antibody library for identification. **D** Overall, 92 recombinant phage clones were randomly selected from the third round of panning and added to microplates coated with BRV. HRP-conjugated anti-M13 monoclonal antibody was used to detect the bound phages. M13K07 helper phage and PBS served as the negative control and blank control, respectively. Positive clones were defined as those with a sample-to-negative control ratio (P/N) ≥ 2.1, i.e., clones above the reference line were identified as positive.

To amplify the VHH gene, PBMCs were first isolated from the whole blood of the immunized Bactrian camel, and total RNA was extracted and reverse transcripted to complementary DNA (cDNA). The cDNA served as the template for the first-round PCR, which targeted variable region gene sequences including the leader signal peptide. This step successfully generated amplicons of approximately 900 bp (VH–CH1–CH2) and 600 bp (VHH–CH2) (Figure 2A). The 600-bp fragment, covering the region from the leader peptide to the CH2 domain, was gel-purified and used as the template for the second-round PCR. This subsequent amplification produced the full-length VHH gene (spanning FR1 to FR4) with a size of approximately 400 bp (Figure 2B).

For the construction of a VHH phage display library, the digested VHH fragments were ligated into the similarly digested pMECS vector using T4 DNA ligase. The insertion efficiency of the VHH gene into the recombinant pMECS–VHH plasmid was assessed by colony PCR. Among 16 randomly selected clones, 13 showed bands of approximately 750 bp, consistent with the expected size, indicating a recombination rate of 81.25% (Figure 2C). The library then underwent three rounds of biopanning against BRV, during which BRV-specific phage clones were enriched by 67.97-fold in the third round (Additional file 3).

To identify BRV-binding clones, the enriched phage library was screened by phage ELISA. The OD_450_ nm value of the negative control was 0.2245, and samples with OD_450_ nm values ≥ 2.1 times that of the negative control were considered positive. A total of 42 positive clones were identified (Figure 2D). From these, clones exhibiting higher binding signals were selected for sequencing. On the basis of sequence verification and high binding activity, clones 7, 73, and 93 were chosen for further functional characterization.

### Expression and purification of BRV-specific sdAbs

**Figure 3:**
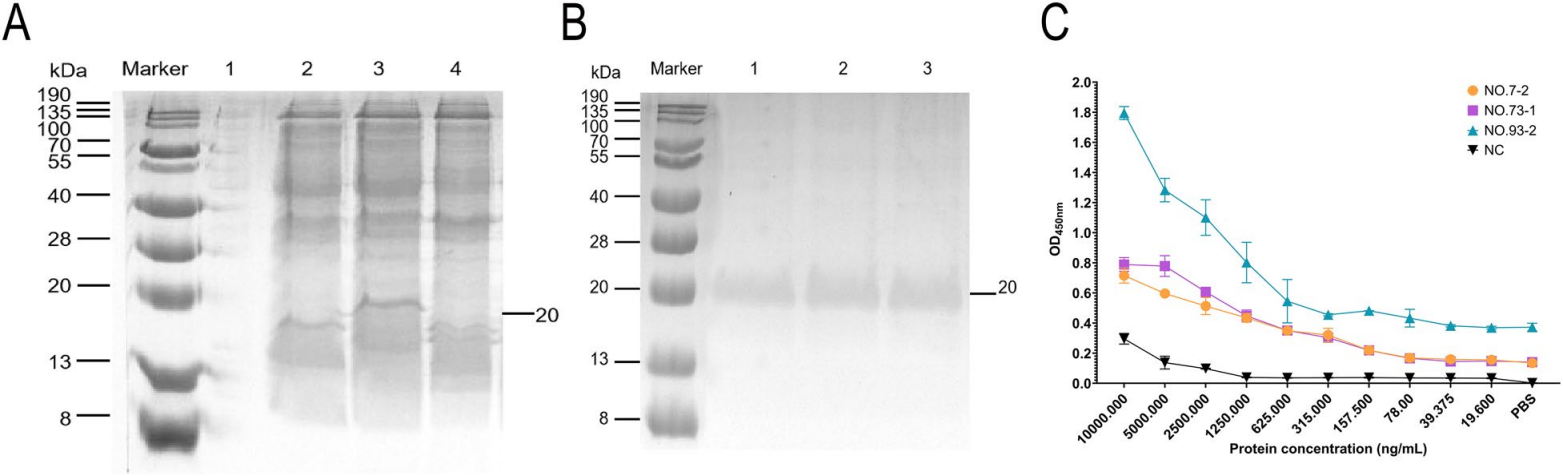
**Preparation of BRV-specific sdAbs and detection of binding activity by ELISA**. **A** Recombinant anti-BRV VHH antibodies were induced in *E. coli*; lane marker represents the protein marker (8–190 kDa), lane 1 shows the bacterial lysate before induction, lane 2 shows the bacterial lysate after induction, lane 3 shows the supernatant of the bacterial lysate, and lane 4 shows the pellet of the bacterial lysate. **B** The collected supernatant of the bacterial lysate was purified using Ni–NTA Sefinose resin to obtain recombinant anti-BRV VHH antibodies. Lane marker represents the protein marker (8–190 kDa), lanes 1–4 show the anti-BRV VHH antibodies. **C** Indirect ELISA was used to detect the binding activity of recombinant VHH clones 7, 73, and 93 to BRV. The purified protein from pET–22(+) empty vector-transformed bacteria was used as the negative control.

To produce BRV-specific sdAbs, the VHH gene was subcloned into the pET–22b(+) expression vector and transformed into *E. coli* BL21 (DE3) competent cells for prokaryotic expression. SDS–PAGE analysis of the soluble fraction from the cell lysates revealed a distinct protein band of approximately 20 kDa (Figure 3A), indicating successful soluble expression of the recombinant VHH protein. Further purification yielded a single predominant band at the expected molecular weight (~20 kDa) with no notable contaminating bands, confirming the high purity of the isolated VHH antibody (Figure 3B).

### In vitro functional activity of BRV-specific sdAbs

#### Binding activity by ELISA

To evaluate the binding activity of the three sdAbs clones, an indirect ELISA was performed against BRV antigens. All three clones exhibited concentration-dependent binding to BRV, with effective antigen recognition maintained even at a low concentration of 625 ng/mL (Figure 3C).

#### Immunofluorescence analysis of BRV-specific sdAbs

**Figure 4:**
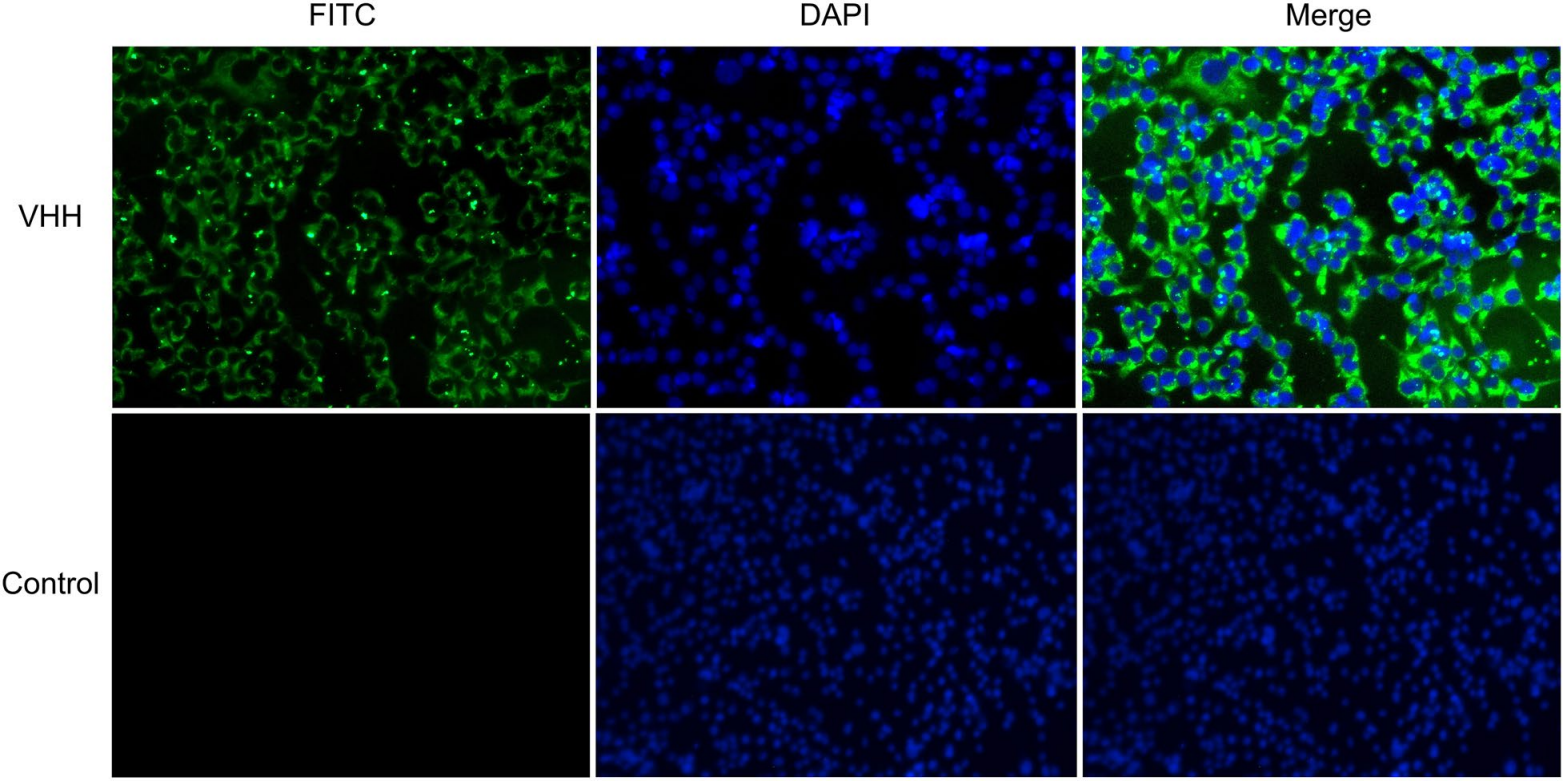
**Detection of binding activity of specific VHH antibodies by cellular immunofluorescence assay**. Cellular immunofluorescence assay for the binding of recombinant clone 73 to BRV.

The binding specificity of the recombinant VHH to BRV-infected cells was further assessed by indirect immunofluorescence assay. Representative results from clone 73 are shown in Figure 4. At 488 nm excitation, distinct green fluorescence was observed in BRV-infected MA-104 cells incubated with clone 73, whereas no specific signal was detected in the control group. These results demonstrate that the purified sdAbs specifically bind to BRV during infection and replication in host cells.

#### Virus neutralization test of BRV-specific sdAbs

**Figure 5:**
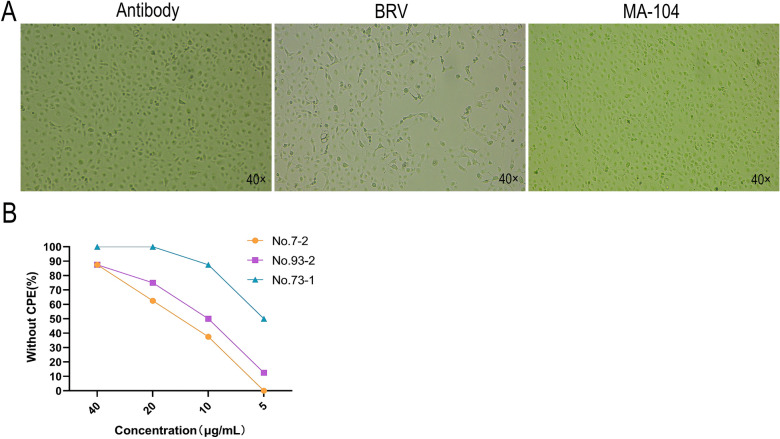
**Detection of neutralizing activity of specific sdAbs**. Virus neutralization test was used to detect the neutralizing activity of sdAbs: **A** BRV pretreated with trypsin was co-incubated with sdAbs at serial concentrations and then used to infect MA104 cells. **B** The optimal neutralizing concentration of sdAbs against BRV was determined by virus neutralization. Recombinant sdAbs clones 7, 73, and 93 could neutralize BRV at concentrations of 40 μg/mL, 20 μg/mL and 20 μg/mL, respectively.

Following confirmation of the specific binding of the sdAbs to BRV, we further evaluated their ability to neutralize viral infection in vitro. MA-104 cells were treated with purified sdAbs clones 7, 73, and 93, with control groups consisting of BRV-infected cells and normal cells. The cells were continuously monitored under an inverted microscope for 3 days, with examinations conducted at 12-h intervals until CPE occurred, and images were manually examined. The results demonstrated that when BRV was co-incubated with the antibodies, no CPE was observed in the antibody-treated groups (Figure 5A). In contrast, the BRV-infected control group exhibited significant CPE, characterized by spindle-shaped or irregular cell morphology, cell shrinkage, widened intercellular spaces, and cell detachment (Figure 5A). Normal MA-104 cells maintained their typical morphology (Figure 5A). Based on lesion count calculations, recombinant clones 7, 73, and 93, at concentrations ≥ 40 µg/mL, ≥ 20 µg/mL, and ≥ 20 µg/mL, respectively, could effectively neutralize ≥ 50% of BRV-infected cells (Figure 5B).

### Protective efficacy in the calf challenge model

#### Clinical score assessment

**Figure 6:**
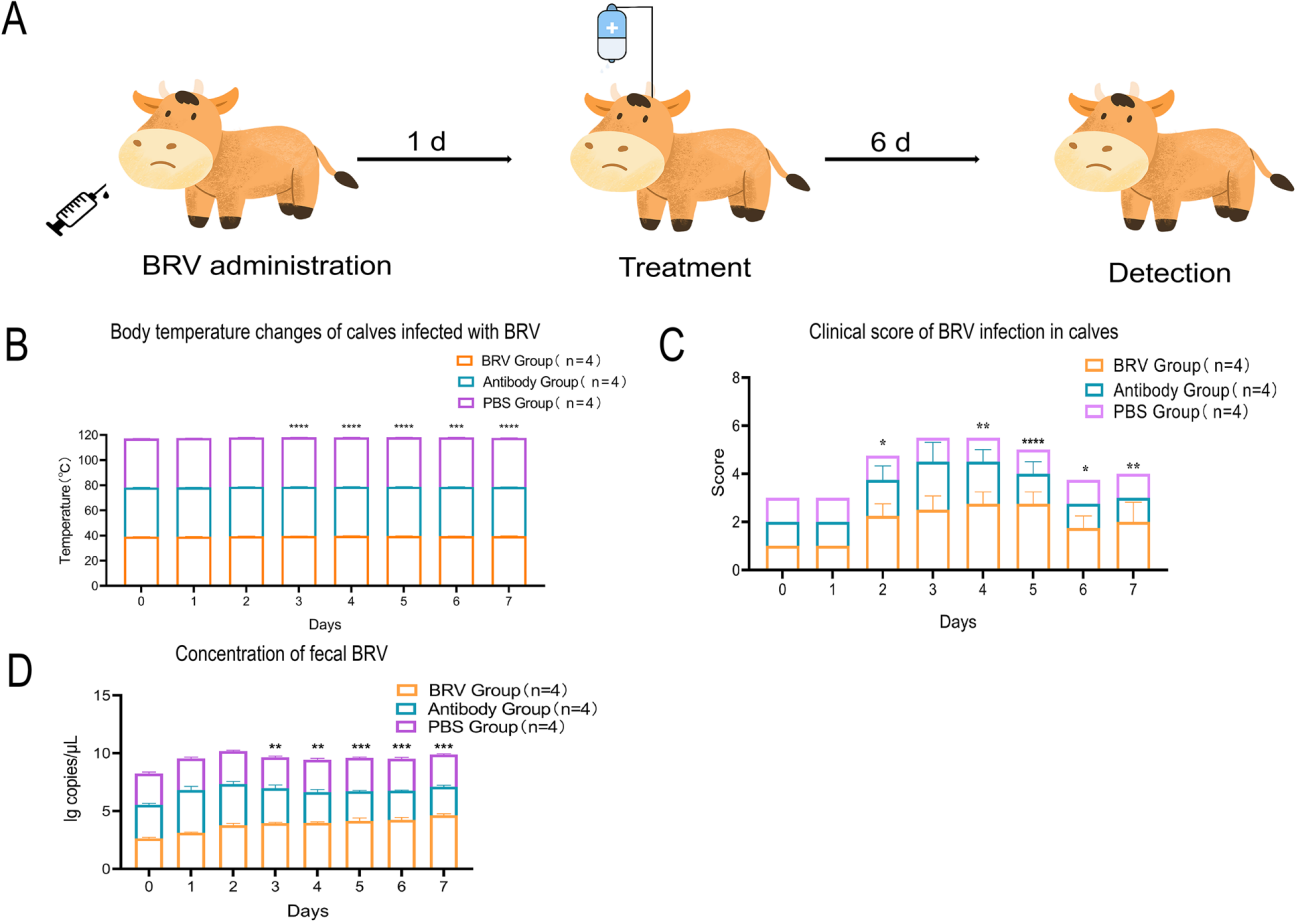
**BRV challenge test in calves and therapeutic efficacy of recombinant sdAbs**. **A** After oral administration of BRV, recombinant sdAbs was intravenously injected for six consecutive days, and its efficacy was evaluated. **B** The body temperature changes of BRV-infected calves were shown by measuring rectal temperature. The horizontal axis represents days, and the vertical axis represents body temperature. **C** The clinical scores of BRV-infected calves are presented. The horizontal axis is days, and the vertical axis is clinical scores. **D** The concentration of BRV in calf feces was detected by RT-qPCR. The horizontal axis represents days, and the vertical axis represents the concentration of BRV in feces.

On the basis of the promising in vitro neutralization results, we further assessed the protective efficacy of the candidate antibody in a calf model of BRV-induced diarrhea. Calves were assigned to three groups: BRV-infected, PBS control, or antibody-treated (Figure 6A). Subsequently, 1 day after oral BRV challenge, antibodies were administered intravenously, and clinical observations were recorded. Calves in the BRV-infected group developed high fever starting on day 2 post-inoculation, which peaked on day 4 before gradually declining; however, their body temperatures remained consistently higher than those in the other two groups throughout the observation period (Figure 6B). All BRV-infected calves exhibited severe diarrhea, characterized by watery yellow stools, accompanied by lethargy, reduced feed intake, and recumbency in some cases (Figure 6C). Necropsy revealed intestinal wall edema and congestion, presenting as dark-red coloration. In contrast, calves receiving antibody treatment displayed only transient soft stools and mild hyporexia during the early stage of infection. Following sdAbs administration, clinical conditions improved markedly: watery diarrhea began to resolve by day 2 post-treatment, and stools normalized by day 4. Mental status and feeding behavior also recovered to baseline levels within 4 days after antibody intervention (Figure 6C). At necropsy, only minor intestinal petechiae were observed, with normal wall color (pale pink) and thickness. Calves in the PBS control group maintained normal body temperature and clinical scores throughout the study, showing no signs of disease (Figure 6B–C).

#### Viral load analysis

Rectal swab samples were collected from each group of calves for viral RNA extraction, followed by RT-qPCR to determine viral load. Absolute quantification of viral RNA was performed using a standard curve-based method. The standard curve was generated using tenfold serial dilutions of a standard containing the target gene fragment, with the regression equation:

$$C_{q} = - 3.1519 \times \log_{10} \left( {copies} \right)\, + \,40.692$$

The amplification efficiency of the assay was 107.6%, and the linear correlation coefficient was *R*^2^ > 0.99. Viral RNA copy numbers were calculated from the Cq values according to this equation to evaluate viral shedding levels in different groups of calves. As shown in Figure 6D, the fecal viral loads differed significantly among the three groups. In the BRV-infected group, high viral loads were consistently detected from day 2 to day 7 post-challenge. In the antibody-treated group, viral load increased gradually after challenge; however, from day 3 onward, it was significantly lower than that in the BRV-infected group (*p* < 0.01 to *p* < 0.001), indicating effective suppression of viral replication by the antibody. In the PBS control group, viral RNA remained undetectable throughout the experimental period, with the lowest values observed across all samples.

#### H&E staining results

**Figure 7:**
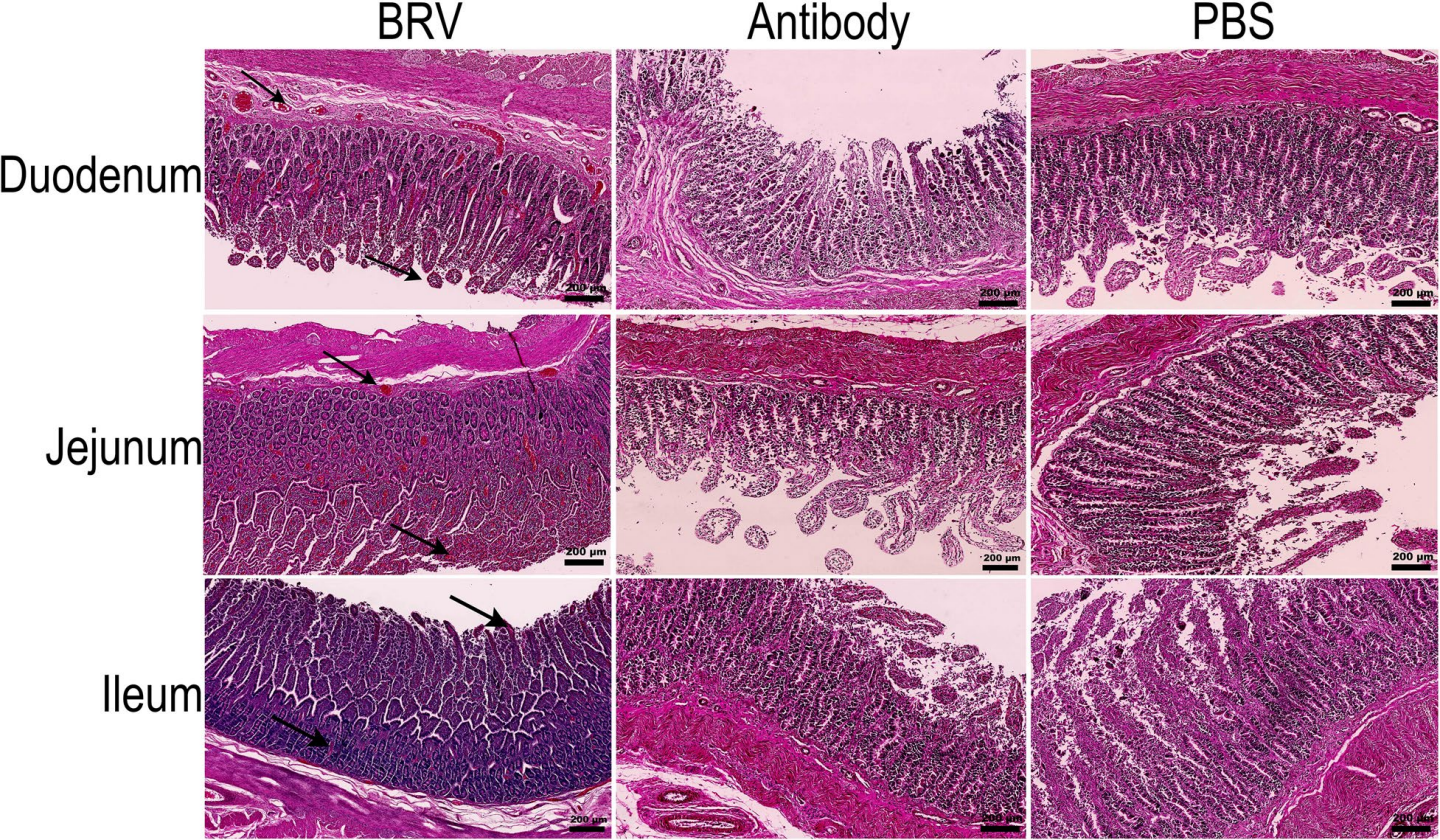
**Histopathological changes in the small intestinal tissues of BRV-infected calves**. H&E staining was used to observe the histopathological changes in the duodenum, jejunum, and ileum of calves in the BRV group, antibody group, and PBS group (scale bar = 200 μm).

To further evaluate the protective effect of the antibody against BRV infection at the histopathological level, H&E staining was performed on small intestinal tissues collected from calves in each group. The BRV-infected calves exhibited marked intestinal damage across multiple segments, characterized by villous atrophy and shortening in the ileum, lymphoid follicle hyperplasia, enhanced lymphocyte infiltration, and the presence of hemorrhagic foci throughout the intestinal tissues as shown in Figure 7. In contrast, the antibody-treated group showed only focal villous blunting and detachment, with no evidence of inflammatory cell infiltration or hemorrhage in the submucosa, and overall tissue integrity was substantially preserved compared with the BRV-infected group (Figure 7). The PBS control group displayed no significant pathological alterations (Figure 7).

In summary, comprehensive evaluation based on clinical signs, viral RNA load, and histopathological outcomes consistently demonstrates that antibody treatment effectively mitigates BRV-induced diarrhea in calves.

## Discussion

Calf diarrhea poses a significant threat to the cattle industry, with BRV being one of the leading causative agents in large-scale farms worldwide. Currently, nine commercial BRV vaccines are available internationally; most of them are multivalent vaccine formulations targeting multiple pathogens [25]. The majority are inactivated vaccines, supplemented by a limited number of live attenuated versions. However, inactivated vaccines carry the potential risk of incomplete inactivation, whereas live attenuated vaccines may revert to virulence. To address these limitations, subunit vaccines have emerged as a promising alternative. Studies have demonstrated that VP7-based empty capsid subunit vaccines exhibit efficacy comparable to that of whole-virus vaccines. Moreover, both the VP7-thermostable enterotoxin fusion protein and multi-copy VP8 chimera have exhibited high immunogenicity in experimental studies [26].

Antibody research against rotavirus (RV) has evolved into a diverse and multi-mechanistic portfolio targeting various viral components. In conventional antibody studies, Jiang et al. demonstrated that intravenous administration of VP4-specific IgG significantly reduced RV infection rates in primate models, with serum IgG titers showing positive correlation with protective efficacy [27]. Li et al. isolated IgA antibodies targeting VP4 from the porcine rotavirus G9P strain (this protein is a truncated form containing the VP8 subunit and the external domain of VP5) and observed a correlation between IgA levels, neutralizing antibody titers, and reduced viral shedding [22]. In addition, the VP6-specific IgA antibodies identified by Burns et al. (e.g., 7D9 and 2C5), although lacking direct in vitro neutralizing activity, can confer protective effects through intracellular mechanisms in mouse models [28].

In the realm of genetically engineered antibodies, Vega et al. isolated VP6-specific VHH from immunized alpacas that demonstrated broad-spectrum neutralization against multiple RV genotypes, including G1P [8] and G2P [4] in vitro [29, 30]. Research on nonstructural proteins has also advanced, with Hou et al. showing that anti-NSP4 monoclonal antibodies can not only reduce the incidence of rotavirus-associated diarrhea in mice but also inhibit NSP4-induced apoptosis of intestinal epithelial cells [31, 32]. Clinically, a systematic review by Patel et al. indicated a correlation between postvaccination serum VP7-IgA titers and protection against RV [33, 34].

Additionally, egg yolk immunoglobulin (IgY) against BRV offers a practical approach for passive immunization [35]. In one strategy, Lohmann Brown Classic laying hens were immunized with BRV antigen, and specific IgY extracted from their egg yolks was orally administered to newborn calves as a milk supplement, demonstrating both protective and immunomodulatory effects against BRV-induced diarrhea [36].

The abovementioned studies have demonstrated that immunotherapeutic strategies against rotavirus involve different antibody types, diverse viral targets, and multiple mechanisms of action, providing various insights for the development of antiviral strategies. On the basis of this research background, the present study further evaluated the potential application value of sdAbs in BRV prevention and control, and experimentally analyzed their binding characteristics and neutralizing capabilities.

Regarding binding activity, the VHH antibodies obtained in this study exhibited marked concentration-dependent binding to BRV antigen in ELISA, and stable signals were still detectable at an antibody concentration of 625 ng/mL. Immunofluorescence assays showed that distinct fluorescence signals were observed in BRV-infected MA-104 cells under 488 nm excitation, whereas no corresponding signals were detected in uninfected control cells.

In in vitro neutralization assays, the VHH antibodies exhibited potent viral inhibitory effects: Clone 73 inhibited ≥ 50% of infected cells at 20 μg/mL, while clone 7 exerted similar effects at ≥ 40 μg/mL. Meanwhile, no typical CPE were observed in MA-104 cells after incubation with the antibodies, whereas significant CPE was present in the control group. The in vivo results provided further support. BRV-infected calves treated with intravenous injection of VHH antibodies exhibited only transient soft feces and recovered normal fecal consistency by approximately day 4. Postmortem examination showed only a small number of petechiae in the intestine, with overall structural integrity preserved. RT-qPCR analysis revealed that viral loads were significantly reduced from day 3 post-infection (*p* < 0.01–0.001), with a shorter duration of viral shedding.

From a production perspective, our VHH antibodies were efficiently expressed in soluble form using the *E. coli* BL21 (DE3) system, showing a clear 20 kDa band in lysates and high purity after purification. This prokaryotic platform is low cost, rapid, and readily scalable. The prokaryotic expression platform demonstrates significant advantages in terms of cost-effectiveness, production efficiency, and scalability, offering a reliable solution for rapid protein production. Meanwhile, other expression systems each possess distinct value and applicable scenarios: PoRV VP4*-specific IgA can be isolated from serum or mucosal secretions, enabling precise targeting, although its preparation process is relatively complex and requires further optimization of batch-to-batch consistency [37]; the baculovirus-insect larvae system used for VP6-specific VHH (clone 3B2) excels in preserving protein activity but currently faces challenges in high costs and scalability for industrial-level production [29, 30]; BRV-specific IgY, purified from immunized eggs, eliminates the need for cell culture systems and simplifies certain operational steps, yet its yield and titer are subject to variability due to avian individual differences, and the process demands considerable labor for purification, with final product quality stability requiring further improvement [35].

## Research conclusions and future perspectives

The BRV sdAbs developed in this study have been experimentally demonstrated to exhibit high antigen-binding affinity and potent neutralizing activity. These sdAbs show considerable promise as therapeutic neutralizing agents, offering a viable strategy to address the current lack of specific treatments for BRV infection in both clinical and market contexts. However, several limitations of this work should be noted: The precise molecular mechanism by which these sdAbs neutralize BRV remains unclear; their efficacy has been assessed against only a single serotype, leaving their cross-neutralizing potential undetermined; and the limited sample size in large-animal trials may affect the statistical reliability of the findings. Consequently, future studies should prioritize elucidating the neutralization mechanism, expanding the evaluation to include multiple BRV serotypes, and validating broad-spectrum efficacy. Further optimization—such as refining phage display screening protocols, increasing the diversity of antibody libraries, and improving antigen purity—could also enhance the expression levels, binding affinity, and clinical potential of these sdAbs.

## Supplementary Information

Additional file 1. **Animal clinical symptom score table**. Calf clinical signs were assessed based on rectal temperature, mental state, appetite, and diarrhea, and classified into four grades accordingly.

Additional file 2. **Serum virus neutralization test.** These results indicate that a 1:21 dilution of camel serum protects 50% of MA104 cells from CPE.

Additional file 3.** The enrichment of phage display library after three round of screening**. After three rounds of iterative screening, BRV-specific phages were enriched with enrichment factors of 1, 14.80, and 67.97, respectively.

## Data Availability

All datasets generated or analyzed during this study are available from the corresponding author upon reasonable request.
