# Supplementary material for: Potent neutralization and therapeutic efficacy of bovine rotavirus-specific VHH antibodies in infected calves
Source: Vet Res. 2026 May 21;57:82. doi: 10.1186/s13567-026-01765-3 (PMC13195868; doi:10.1186/s13567-026-01765-3)
Supplement: Supplementary file 1 — Additional file 1. Animal clinical symptom score table. Calf clinical signs were assessed based on rectal temperature, mental state, appetite, and diarrhea, and classified into four grades accordingly. [file 13567_2026_1765_MOESM1_ESM.docx]

**Additional file 1: Animal clinical symptom score table.**

| Clinical symptoms | Asymptomatic (1) | Mild (2) | Moderate (3) | Severe (4) |
| --- | --- | --- | --- | --- |
| Temperature | <39.5°C | 39.6-40.0°C | 40.1-40.5°C | 40.6-41.5°C |
| Mental State | Normal | Lethargic | Depressed, unwilling to move | Lethargic, unresponsive to stimuli |
| Stool State | Normal | Soft, slightly loose | Watery stool | Watery stool with blood |
| Eating Status | Normal | Slightly reduced | More than half reduced | Not eating |

Rectal temperature: Fever is a primary indicator of systemic infection or inflammation. The thresholds reflect progressive severity, with higher temperatures (>40.5°C) indicating greater systemic involvement. Mental state: Behavioral changes (e.g., lethargy, depression) correlate with worsening illness and neurological/immune responses. Stool state: Diarrhea progression (soft → watery → bloody) reflects gastrointestinal pathology severity, with bloody diarrhea suggesting advanced enteric disease. Eating status: Reduced appetite is a sensitive marker of morbidity; refusal to eat signals critical illness. Table S1 shows the clinical symptom scoring table. The evaluation of clinical symptoms includes measuring rectal temperature, mental state, feeding status, and occurrences of diarrhea. Based on the symptoms described in the table, the clinical symptoms of calves can be classified into four levels.
