# Supplementary material for: Potent neutralization and therapeutic efficacy of bovine rotavirus-specific VHH antibodies in infected calves
Source: Vet Res. 2026 May 21;57:82. doi: 10.1186/s13567-026-01765-3 (PMC13195868; doi:10.1186/s13567-026-01765-3)
Supplement: Supplementary file 2 — Additional file 2. Serum virus neutralization test. These results indicate that a 1:21 dilution of camel serum protects 50% of MA104 cells from CPE. [file 13567_2026_1765_MOESM2_ESM.docx]

**Additional file 2: Serum virus neutralization test.**

These results indicate that a 1:21 dilution of camel serum protects 50% of MA104 cells from CPE.

| Serum dilution | CPE wells/total wells | CPE wells | Cumulative CPE wells | Non-CPE wells | Cumulative non-CPE wells | CPE rate |
| --- | --- | --- | --- | --- | --- | --- |
| 1:2 | 0/8 | 0 | 0 | 8 | 31 | 0/31 |
| 1:4 | 1/8 | 1 | 1 | 7 | 23 | 1/24 |
| 1:8 | 1/8 | 1 | 2 | 7 | 16 | 4/18 |
| 1:16 | 3/8 | 3 | 5 | 5 | 9 | 5/14 |
| 1:32 | 5/8 | 5 | 10 | 3 | 4 | 10/14 |
| 1:64 | 7/8 | 7 | 17 | 1 | 1 | 17/18 |
| 1:128 | 8/8 | 8 | 25 | 0 | 0 | 25/25 |
