# Supplementary material for: Potent neutralization and therapeutic efficacy of bovine rotavirus-specific VHH antibodies in infected calves
Source: Vet Res. 2026 May 21;57:82. doi: 10.1186/s13567-026-01765-3 (PMC13195868; doi:10.1186/s13567-026-01765-3)

**Additional file 3: The enrichment of phage display library after three round of screening.** After three rounds of iterative screening, BRV-specific phages were enriched with enrichment factors of 1, 14.80, and 67.97, respectively.


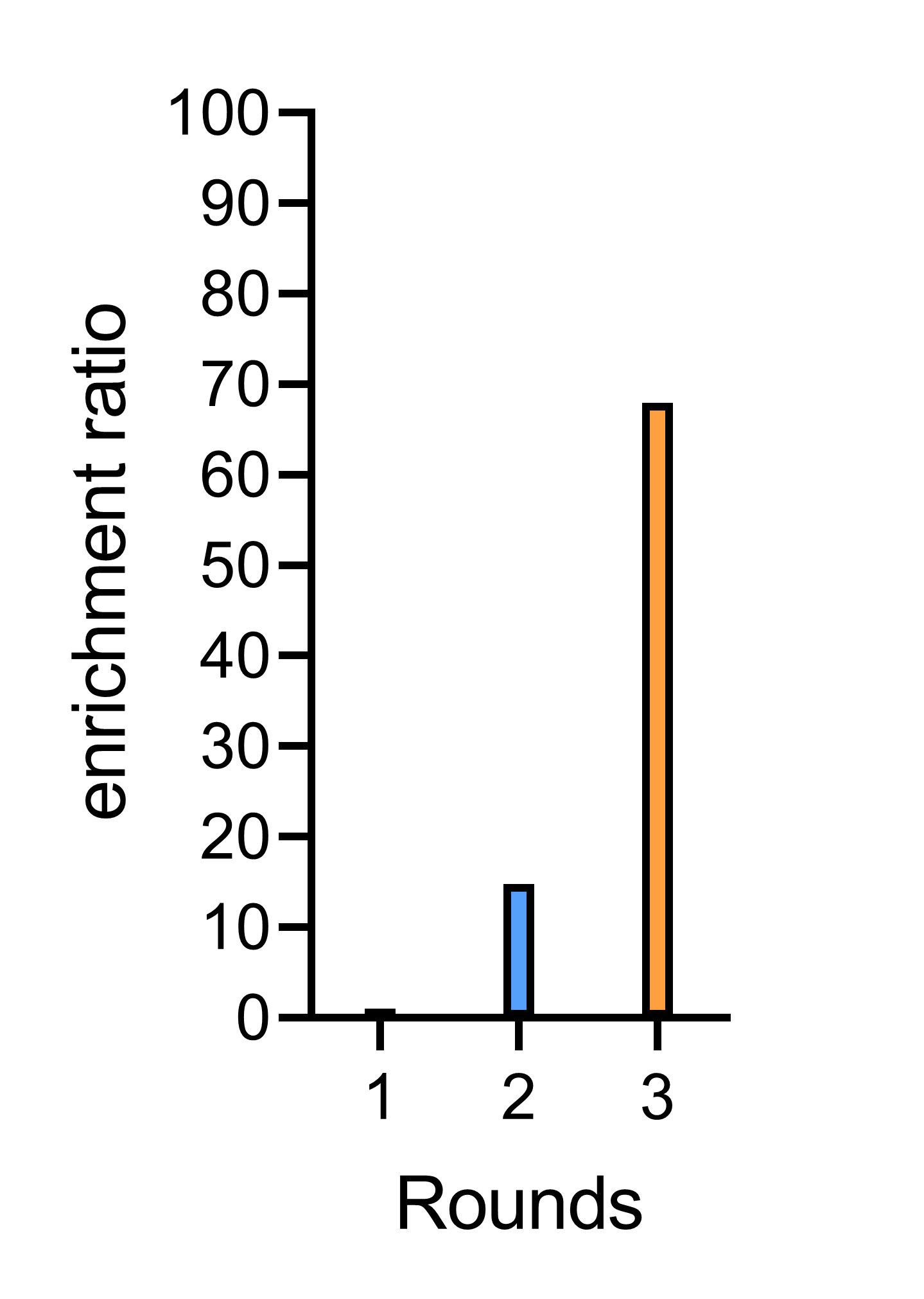

Supplement: Supplementary file 3 — Additional file 3. The enrichment of phage display library after three round of screening. After three rounds of iterative screening, BRV-specific phages were enriched with enrichment factors of 1, 14.80, and 67.97, respectively. [file 13567_2026_1765_MOESM3_ESM.docx]
